# P-1588. Effect of Multiplex Polymerase Chain Reaction (PCR) versus Matrix-Assisted Laser Desorption/Ionization-Time of Flight Mass Spectrometry (MALDI-TOF MF) on Time to Optimal Therapy in Patients with Positive Blood Cultures

**DOI:** 10.1093/ofid/ofae631.1755

**Published:** 2025-01-29

**Authors:** Alexandria Baum, Jessica Miller, Victoria Gavaghan, Jill Argotsinger

**Affiliations:** Advocate Health Care Midwest Region, Wilmette, Illinois; Advocate Lutheran General Hospital, Chicago, Illinois; Advocate Health Care Midwest Region, Wilmette, Illinois; Advocate Lutheran General Hospital, Chicago, Illinois

## Abstract

**Background:**

Rapid diagnostic technology is an important tool that can be used to optimize antimicrobial therapy for patients with bloodstream infections Several technologies for rapid organism identification exist including matrix-assisted laser desorption/ionization-time of flight mass spectrometry (MALDI-TOF MS) and multiplex polymerase chain reaction (PCR). The primary objective is to compare time to optimal antimicrobial therapy with MALDI-TOF MS vs multiplex PCR rapid diagnostic systems for patients with positive blood cultures.

**Methods:**

This multicenter, retrospective, cohort study included patients with a positive blood culture and identification via MALDI-TOF MS (June 1st – June 7th, 2021) compared to multiplex PCR (June 1st – June 7th, 2022). Patients were excluded if expired or discharged before organism identification, transferred from an outside hospital with a positive blood culture, if rapid diagnostics not performed, if identified pathogen had no defined optimal therapy per system guidelines or polymicrobial infection. The primary outcome was time to optimal therapy (TTOT). Secondary outcomes included time to effective therapy (TTET), length of stay, and clinical failure defined as recurrence of bacteremia within 30 days or in-hospital mortality.

**Results:**

A total of 221 patients were included (MALDI-TOF MS = 117; multiplex PCR = 104). Utilization of multiplex PCR compared to MALDI-TOF MS significantly reduced overall TTOT (21.1 hours vs 41.1 hours, p < 0.01). This significant reduction in TTOT with multiplex PCR was observed with gram-negative bacteremia (4 hours vs 37 hours, p < 0.01) and gram-positive bacteremia (17.8 hours vs 41.1 hours, p < 0.01) but was not statistically significant for blood cultures deemed contaminants (42 hours vs 43.5 hours, p=0.58). TTET was improved for multiplex PCR although not statistically significant (1.5 hours vs 7.5 hours, p=0.06). No differences were observed for clinical failure or hospital length of stay.

**Conclusion:**

Multiplex PCR significantly decreased time to optimal antimicrobial therapy when compared to MALDI-TOF MS for patients with positive blood cultures.

**Disclosures:**

**All Authors**: No reported disclosures

